# Low-dose exposure to malathion and radiation results in the dysregulation of multiple neuronal processes, inducing neurotoxicity and neurodegeneration in mouse

**DOI:** 10.1007/s11356-023-31085-4

**Published:** 2023-12-01

**Authors:** Rekha Koravadi Narasimhamurthy, Babu Santhi Venkidesh, Sangeetha Nayak, Dinesh Reghunathan, Sandeep Mallya, Krishna Sharan, Bola Sadashiva Satish Rao, Kamalesh Dattaram Mumbrekar

**Affiliations:** 1 Department of Radiation Biology & Toxicology, Manipal School of Life Sciences, Manipal Academy of Higher Education, Manipal, 576104 Karnataka India; 2 Department of Cell and Molecular Biology, Manipal School of Life Sciences, Manipal Academy of Higher Education, Manipal, 576104 Karnataka India; 3 Department of Bioinformatics, Manipal School of Life Sciences, Manipal Academy of Higher Education, Manipal, 576104 Karnataka India; 4 Department of Radiotherapy, Kasturba Medical College, Manipal Academy of Higher Education, Manipal, 576104 Karnataka India; 5 Directorate of Research, Manipal Academy of Higher Education, Manipal, 576104 Karnataka India

**Keywords:** Malathion, Low-dose radiation, Organophosphates, Neurodegeneration, Coexposure, Neurotoxicity

## Abstract

Neurodegenerative disorders are a debilitating and persistent threat to the global elderly population, carrying grim outcomes. Their genesis is often multifactorial, with a history of prior exposure to xenobiotics such as pesticides, heavy metals, enviornmental pollutants, ionizing radiation etc,. A holistic molecular insight into their mechanistic induction upon single or combinatorial exposure to different toxicants is still unclear. In the present study, one-month-old C57BL/6 male mice were administered orally with malathion (50 mg/kg body wt. for 14 days) and single whole-body radiation (0.5 Gy) on the 8^th^ day. Post-treatment, behavioural assays for exploratory behaviour, memory, and learning were performed. After sacrifice, brains were collected for histology, biochemical assays, and transcriptomic analysis. Transcriptomic analysis revealed several altered processes like synaptic transmission and plasticity, neuronal survival, proliferation, and death. Signalling pathways like MAPK, PI3K-Akt, Apelin, NF-κB, cAMP, Notch etc., and pathways related to neurodegenerative diseases were altered. Increased astrogliosis was observed in the radiation and coexposure groups, with significant neuronal cell death and a reduction in the expression of NeuN. Sholl analysis, dendritic arborization and spine density studies revealed decreased total apical neuronal path length and dendritic spine density. Reduced levels of the antioxidants GST and GSH and acetylcholinesterase enzyme activity were also detected. However, no changes were seen in exploratory behaviour or learning and memory post-treatment. Thus, explicating the molecular mechanisms behind malathion and radiation can provide novel insights into external factor-driven neurotoxicity and neurodegenerative pathogenesis.

**Supplementary Information:**

The online version contains supplementary material available at 10.1007/s11356-023-31085-4.

## Introduction

Neurodegeneration constitutes the progressive deterioration in the morphology and function of neurons of selective brain regions that pathologically manifests in the form of various neurodegenerative disorders, such as Alzheimer’s disease (AD), Parkinson’s disease (PD), and amyotrophic lateral sclerosis (ALS). Emerging studies suggest that the observed increase in neurodegenerative disorders over the years is proportional to the increased exposure to neurotoxic agents (Chin-Chan et al. 2015), with its impact varying in complexity, severity, and clinical significance. Xenobiotics, like various classes of drugs, cosmetics, pesticides, food additives, industrial chemicals, environmental pollutants, etc., are some common chemicals that individuals can get exposed to in their lifetime (Patterson et al. 2010). Among these etiological factors, xenobiotics such as pesticides and ionizing radiation (IR) are at the forefront, mainly due to their increasing application (Walther et al. 2023). Pesticides have been linked to neurodegeneration through oxidative stress, mitochondrial dysfunction, DNA damage and altered cellular signalling (Narasimhamurthy et al. 2022a; Rodrigues et al. 2022; Prathiksha et al. 2023), while IR has also been studied for their role in inducing neurotoxicity (Sharma et al. 2018).

Malathion is a broad-spectrum organophosphate used mainly for agricultural, domestic, and public health purposes, and owing to its slightly less toxic nature, it is extensively and sometimes recklessly overused in several parts of the world (Badr 2020). The route of exposure can be occupational (farmers, pest control industries, production), household or through the consumption of contaminated food. In non-target organisms, the main mechanism of action of malathion is through the inhibition of acetylcholinesterase (AchE) activity in the brain, leading to adverse effects such as decrease in the strength of the cranial motor nerve, proximal limb muscle, neck flexors, restlessness, hyperexcitability, seizure and, in extreme cases, death (Tchounwou et al. 2015). In addition, chronic exposure to malathion is known to generate free radicals and cause oxidative stress (Fortunato et al. 2006) and DNA damage (100 and 150 mg/kg bw) (Réus et al. 2008) in rodent brains. Malathion also causes neuroinflammation by the activation of microglia leading to neuronal death (50, 100 and 200 mg/kg bw) (Ahmed et al. 2017) and increases pro-inflammatory cytokines such as TNF-α (Tumor necrosis factor α) and IL-6 (Interleukin 6), which mediates neuronal damage (100 mg/kg bw) (Mohammadzadeh et al. 2018), resulting in cognitive impairment. Furthermore, it interferes with axonal transport via the alteration of motor proteins (Naughton and Terry 2018) and induces mitochondrial dysfunction by altering the levels of mitochondrial complex activities, generation of ROS, ATP depletion, DNA fragmentation, and apoptosis (Venkatesan et al. 2017) in rodents and neuronal cells. Studies have reported the detection of malathion well above the permissible limits in many food items at concentrations as high as 18.26 mg/kg (Hamid et al. 2020), thus, making it a matter of great concern and a point of public interest. Moreover, these exposures are implicated in the aetiology of neurodegenerative-like effects, which call for a deeper understanding of their mechanisms.

Medical usage of IR contributes approximately 98% to the total population dosage through artificial sources while representing 20% of the total population exposure (WHO 2023), making it a public concern. Repeated radio imaging procedures can lead to exposures of up to 0.1 Gy, and there are instances where normal, non-targeted tissues may be exposed to radiation during radiotherapy, with doses ranging from milli to several Gy. For example, a study measuring the out-of-field dose to the hippocampus from radiotherapy procedures measured a maximum of 154.8 mGy reaching the hippocampus (Auerbach et al. 2023). Another study showed that the organ equivalent dose in brain from combined PET/CT carried out in males was 36.9 ± 3.6 mSv and 32.8 ± 5.5 mSv in females (Quinn et al. 2016). Lower doses of IR exposure can cause neurotoxicity by impairing neuronal plasticity, signal transduction, changes in oxidative phosphorylation, altered mitochondrial complex enzyme activity, and impaired antioxidant levels (Narasimhamurthy et al. 2022b). Studies have also reported that IR exposure can induce gene expression changes of several genes crucial for immune response, DNA repair, cell adhesion, and death (Katsura et al. 2016).

There is a huge lack of understanding on how xenobiotics interact in a coexposure, and low-dose setting. Low-dose xenobioticsare generally less adverse, and can have a large detrimental impact on human health when combined with other chemicals (Silins and Högberg 2011). Both malathion and IR have been known to cause DNA damage through the generation of free radicals and decreased levels of antioxidant enzymes leading to apoptosis, with several reports showing that they induce neurotoxicity. Our study investigates the effect of pesticide exposure in younger animals, as children, particularly due to their developing nature, are susceptible to greater damage. This is mainly due to their larger life expectancy and higher chances of latent long-term neurotoxic changes occurring later in their lives, which is not thoroughly understood. In the public scenario, children are distinctively vulnerable to inadvertently ingesting malathion through diet or frequent hand-to-mouth activity, leading to often larger doses to be ingested. Furthermore, malathion is predicted to play a role in the etiology of neurodegenerative diseases; however, its noncholinergic mechanism at the level of pathways is still elusive, and several mechanistic studies conducted thus far have been primarily in in vitro models.

The present study elucidates the mechanism behind pesticide/IR exposure-induced neurotoxicity by employing high-throughput methods using a mouse model. We also correlate how exposure to these agents at a young age can drive the organism toward neurodegenerative outcomes by altering several pathways responsible for neurodegeneration. Furthermore, we also show how these two commonly acting xenobiotics may act in cases of combined exposure, which is especially relevant when applied to a large population.

## Material and methods

### Animals and treatment conditions

C57BL/6 male mice were obtained from the Central Animal Research Facility, Kasturba Medical College, Manipal Academy of Higher Education, Manipal, after duly obtaining ethical clearance from the Institutional Animal Ethics Committee (IAEC/KMC/108/2019). The animals were maintained in polypropylene cages under optimum temperature (20 °C ± 2 °C) and light conditions (10 h light and 14 h dark cycle). Sterile food and water were provided ad libitum*.* Four- to 5-week-old animals weighing approximately 27.05 g ± 0.82 were used for the experiment, and body weight was monitored daily throughout the treatment. Thirty-six animals were divided into four groups containing nine animals each, namely, control, malathion (50 mg/kg malathion orally, approximately 14 times the acceptable daily dose declared by WHO), IR (0.5 Gy), and coexposure (50 mg/kg malathion orally + 0.5 Gy IR). Commercially procured malathion (MP Biomedicals, India) with 96% purity was administered orally in saline through gavage for 14 days continuously, and on the 8^th^ day, a single whole-body exposure to 0.5 Gy of X-ray radiation was delivered on a Versa-HD Linear Accelerator (Elekta, Sweden) using 6 MV Photons. The Integrated Risk Information System (IRIS) Chemical Assessment Summary from the U.S Environmental Protection Agency reported that the dose of 50 mg/kg was the lowest neurological level at which neurological consequences like acetylcholinesterase and reduced body weight were reported in mice. We chose 14-day exposure as a sub-chronic exposure duration to study the early and late effects. The current study deals with the neurological effects at younger ages and looks for the early changes manifested. Furthermore, the duration was also limited to account for the variation in the age and lifespan between mice and humans (Dutta and Sengupta 2016). After the completion of treatment, the animals were subjected to behavioural assays for 5 days, followed by euthanization by cervical dislocation and tissue collection for histological, biochemical, and molecular analysis.

### Open field test for anxiety-related behaviour

This test is mainly performed to assess anxiety-related behaviour and locomotion in mice. The animal was placed in the centre of an open square field facing the side away from the experimenter and allowed to explore for 5 min, which was video-recorded. After 5 min, the animal was removed, and the field was cleaned using 70% alcohol and allowed to dry. The video was then blind-scored for the time spent in the centre (Seibenhener and Wooten 2015) using ANY-maze (Stoelting, USA).

### Novel Object Recognition Test for recognition memory

The test was performed as described previously to analyse recognition memory in mice (Leger et al. 2013). Briefly, animals were habituated to the testing arena for 10 min on day 1. Familiarization was performed, allowing a minimum 20-s exploration of two identical objects followed by the retention phase 24 h later with one of the objects replaced. The experiment was video-recorded and blindly scored, and parameters such as the duration and frequency of exploration of the novel and the familiar object were noted. The discrimination index and recognition index were calculated from the data obtained. The discrimination index (DI) was calculated according to the following formula: (time exploring the novel object — time exploring the familiar object)/(time exploring novel + familiar) * 100, and the recognition index (RI) was calculated according to the following formula: time spent exploring the novel object/total time exploring both objects.

### Sholl analysis and dendritic arborization by Golgi-Cox staining

Anaesthetized mice were intracardially perfused, and the brain was dissected and stored in Golgi-Cox solution for 10–14 days. Using a vibratome (5100 mz, Campden Instruments, England), sections of 150 µm thickness were taken, immersed in 6% sodium carbonate for 20 min, dipped in distilled water, immersed in 70%, 90%, and 100% alcohol for ten, fifteen and 20 min each followed by xylene for 20 min and mounted (Narayanan et al. 2014). Images were captured using an Olympus BX51 (Olympus, Japan) microscope. Neurons were traced using the Simple Neurite Tracer plugin in Fiji software (Schindelin et al. 2012) and Sholl analysis was performed on the traced neurons using the Sholl analysis plugin (Ferreira et al. 2014). The total number of average intersections, the number of intersections at different distances from the soma, and the total path length in both apical and basal neurons were calculated. Furthermore, the spine density in every 10 µm of dendrite was also calculated.

### Nissl staining for neuronal survival

Nissl staining was performed according to the protocol described previously (Venkidesh et al. 2023). Briefly, slides were placed on a slide warmer at 55 °C to melt the paraffin for approximately 20–30 min. Slides were then immersed in xylene twice, followed by twice in 100% alcohol (2 min each), 95% and 75% alcohol (2 min each), and dipped in distilled water (2 min). The slides were stained with cresyl violet (1–2 min) and distilled water (3 min). The previous alcohol and xylene steps were then repeated in reverse and mounted. The images were captured using an Olympus CKX53 (Olympus, Japan) microscope, and image analysis was performed using the Fiji software plugin Cell counter to score the pyknotic cells. Three animals per group, with three sections per animal, were analysed.

### Immunohistochemistry for mature neurons and astroglial activation

Immunohistochemistry was performed as previously described (Venkidesh et al. 2023). Briefly, slides were placed on a slide warmer at 60 °C, placed in two changes of xylene, 100% ethanol two times, 95% ethanol two times (10 min each), and distilled water (5 min). Antigen retrieval was performed in 1 × sodium citrate buffer for 30 min, followed by washing using PBS containing 0.005% Tween-20. Blocking was then performed for 2 h at room temperature in 10% normal serum containing 1% BSA in PBS. Following blocking, the primary antibody dissolved in 1% BSA in PBS according to the respective dilution was applied and incubated in a humidified chamber overnight at 4 °C. NeuN (1:500 dilution, Invitrogen, USA) and GFAP (1:2000 dilution, Invitrogen, USA) markers were used for mature neurons and astroglia, respectively. The following day, after three washes (10 min each), 3% H_2_O_2_ was added to block endogenous peroxidase activity for 15 min in the dark. Following three washes (10 min each), secondary antibody (1:1000 dilution, goat anti-rabbit) (Jackson ImmunoResearch Laboratory, USA) was applied at a dilution of 1:1000 for 1 h at room temperature. The sections were then washed and stained with DAB chromogen, counterstained with hematoxylin, subjected to several upgrades of alcohol and xylene and mounted. Three animals per group (three sections per animal), with two regions per section were considered for scoring. Within each region, a minimum of three images were captured for the analysis. The images were captured using Olympus CKX53, and image analysis was performed using the Fiji plugin, Cell counter. Furthermore, the expression of NeuN was represented as % positively expressed area (Insausti et al. 2015).

### AChE inhibition assay

Modified Ellman’s assay was conducted to assess the levels of acetylcholinesterase inhibition (Banasik et al. 2003). The rate of hydrolysis of acetylthiocholine iodide catalyzed by cholinesterases in tissue was determined colorimetrically with a reagent that detects sulfhydryl groups. Acetylthiocholine iodide was incubated with the sample and the coloured reagent 5,5-dithiobis-2-nitrobenzoic acid (DTNB). The thiocholine produced by the hydrolysis of the substrate reacts with DTNB to form a yellow-coloured anion known as 5-thio-2-nitrobenzoate, the formation of which, was kinetically followed at 412 nm. The absorbance/min was calculated using the slope, and the rate of the reaction was calculated using the formula:

$$\mathrm{Rate }=\left(\frac{\mathrm{Absorbance}}{\mathrm{minute}}\right)/ 1.36\mathrm{\;\times\;}{10}^{4},$$

where 1.36 × 10^4^ is the extinction coefficient of the yellow anion (5-thio-2-nitrobenzoic acid)

Furthermore, the treatment groups were normalized to the control and expressed as percentage normalized activity with respect to the control.

### GSH and GST assay

The intracellular levels of glutathione (GSH) and glutathione-s-transferase (GST) were determined by protocols as described previously (Das et al. 2016). The reduced form of GSH reacts with DTNB to give rise to TNB, which was photometrically measured at 412 nm. GST assay was measured by observing the conjugation of 1-chloro, 2,4-dinitrobenzene (CDNB) with reduced glutathione by observing an increase in the absorbance at 340 nm. One unit of enzyme was conjugated to 10.0 nmol of CDNB with reduced glutathione per minute at 25 °C. The whole brain was homogenized in lysis buffer and utilized for further analysis for the assay. The GST values were calculated by finding the delta absorbance/min at 340 nm and dividing it by the molar extinction coefficient. The GSH and GST values were normalized to the respective protein values measured through the Bradford assay and expressed as μM/mg protein.

### mRNA isolation, cDNA conversion, library preparation, and sequencing

The hippocampus was isolated from freshly dissected mouse brains (2 mice per group) on ice, weighed, and then homogenized with TRIzol (Ambion, USA). The RNA was isolated according to the manufacturer’s protocol, and quality was checked by running on a gel. mRNA isolation and purification were performed using the Dynabead mRNA DIRECT Purification Kit (Invitrogen, USA), and cDNA conversion, amplification, and library preparation were performed using the Ion Total RNA-Seq Kit v2 (Invitrogen, USA) according to the manufacturer’s protocol. Template-positive ion sphere particles (ISPs) were generated using the Ion PI Hi‑Q OT2 200 Kit (Thermo Fischer, USA). Sequencing was carried out using 200 bp sequencing chemistry in an Ion Proton semiconductor sequencing using an Ion PI chip according to the manufacturer’s instructions (Invitrogen, USA). Since the animals were grouped after well defining their population, with matched characteristics like age, sex, weight, and the experimental design is simple with minimum technical and biological variability within the groups, two samples showing high-quality RNA with a substantial yield were selected for analysis in each group.

### Bioinformatic analysis

The transcriptomic data in fastq files was analysed using nf-core/RNAseq pipeline version 3.10.1 on Nextflow version 22.10.4 (Ewels et al. 2020). The quality of the raw fastq files was assessed using FastQC and reported using MultiQC (Ewels et al. 2016). The reads were mapped to the mouse genome (GRCm38) using STAR aligner (Dobin et al. 2013), and the counts were extracted using featureCounts software. Differential expression (DE) analysis between groups was performed using EdgeR (Robinson et al. 2010), an R Bioconductor package. A *p*-value of < 0.05 and fold change of ± 1.5 were defined to establish significant upregulated and downregulated genes, respectively.

The significant differentially expressed genes (DEGs) were then subjected to gene ontology enrichment analysis and pathway enrichment analysis using the online modules in the SR plot (https://www.bioinformatics.com.cn/en). A STRING network (Szklarczyk et al. 2023) to visualize the functional interaction of the DEGs in each treatment group was constructed at a high confidence score (0.7), and only the significant and connected nodes within our DEG network were visualized. The network was imported into Cytoscape (Shannon et al. 2003) and modified using cytoHubba (Chin et al. 2014) to represent the network interaction of the top 50 genes.

### Statistical analysis

Statistical analysis was carried out using GraphPad Prism v.8 software and one-way ANOVA followed by Tukey’s multiple comparisons post hoc test. Data are expressed as the mean ± SD or mean ± SEM, and a *p*-value less than 0.05 was considered significant.

## Results

### Neuronal death

**Fig. 1:**
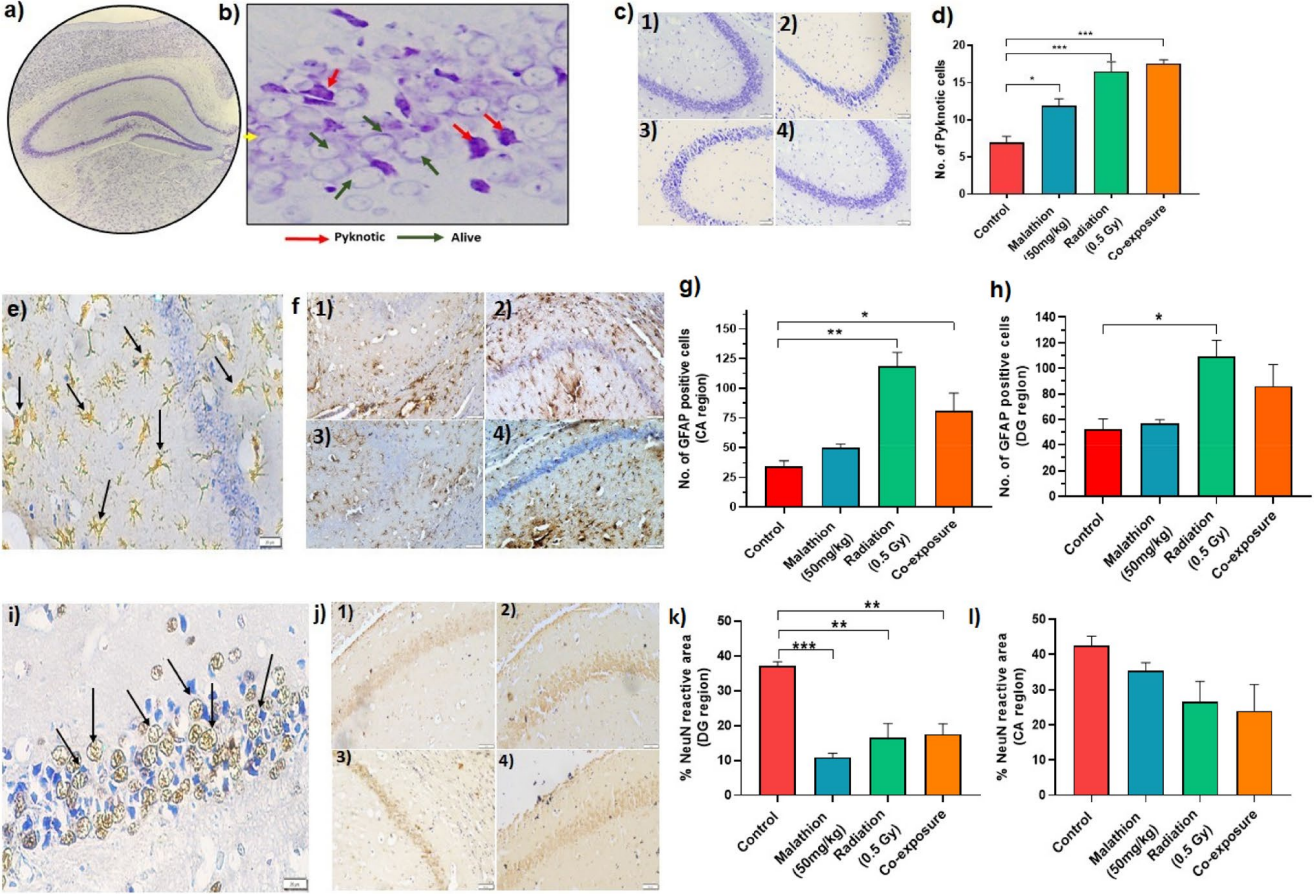
Effect of IR and malathion single and coexposure on neuronal survival, inflammation and maturation. (**a**) Nissl-stained mouse hippocampus; (**b**) Representative image showing pyknotic and live neurons in the mouse hippocampus (40 ×); (**c**) Representative images of hippocampal regions of different groups (20 ×)- 1) Control 2) IR 3) Malathion 4) Coexposure; (**d**) Graph depicting the number of pyknotic neurons in different groups . (**e**) Representative image showing reactive astroglia (40 ×); (**f**) Representative images of GFAP-positive cells in the hippocampus of different groups (20 ×): 1) Control, 2) IR, 3) Malathion, and 4) Coexposure; (**g**) Graph depicting the number of reactive astroglia in the CA region; (**h**) Graph representing the number of reactive astroglia in the DG region. (**i**) Representative image showing NeuN-positive cells (40 ×); (**j**) Representative images of NeuN-positive cells in the hippocampus of different groups (20 ×)- 1) Control 2) IR 3) Malathion 4) Coexposure; (**k**) Graph depicting the percentage of NeuN-positive area in the DG region; (**l**) Graph representing the percentage of NeuN-positive area in the CA region (arrow marks in fig. e and i indicate immunopositive cells)(*n* = 3, *p* < *0.05, **0.01, ***0.001, data represented as ± SEM).

Pyknotic cells were found to be increased in the IR (*p* < 0.001) and malathion (*p* < 0.05) groups as well as in the coexposure group, with the highest number of pyknotic cells in the coexposure group (*p* < 0.001) (Fig. 1a–d). Therefore, coexposure enhanced the effect by inducing the highest number of pyknotic cells, indicating that the combined effect displayed synergism.

### Astroglia activation and neuronal maturation

The glial cell projections were higher in number and increased in thickness in the treatment groups (Fig. 1e, f). In the cornu ammonis (CA3-CA2), the expression of reactive GFAP increased the most in the IR (*p* < 0.01), followed by coexposure groups (*p* < 0.05) (Fig. 1g). In the dentate gyrus (DG) region, increased astrogliosis was observed only in the IR group (*p* < 0.05) (Fig. 1h). NeuN expression decreased the most in the malathion (*p* < 0.001) group, while expression levels in IR (*p* < 0.01) and coexposure groups (*p* < 0.01) (Fig. 1i–k) in the DG region remained comparable, indicating a decreased number of mature neurons in the hippocampal region. This suggests that IR and malathion induced inflammation through the activation of glial cells and impaired the maturity of neurons within the hippocampus. However, the effect in the coexposure group displayed antagonistic interaction with the expression levels falling between malathion and IR alone groups or in comparable levels of the IR group.

### Dendritic arborization and spine density

**Fig. 2:**
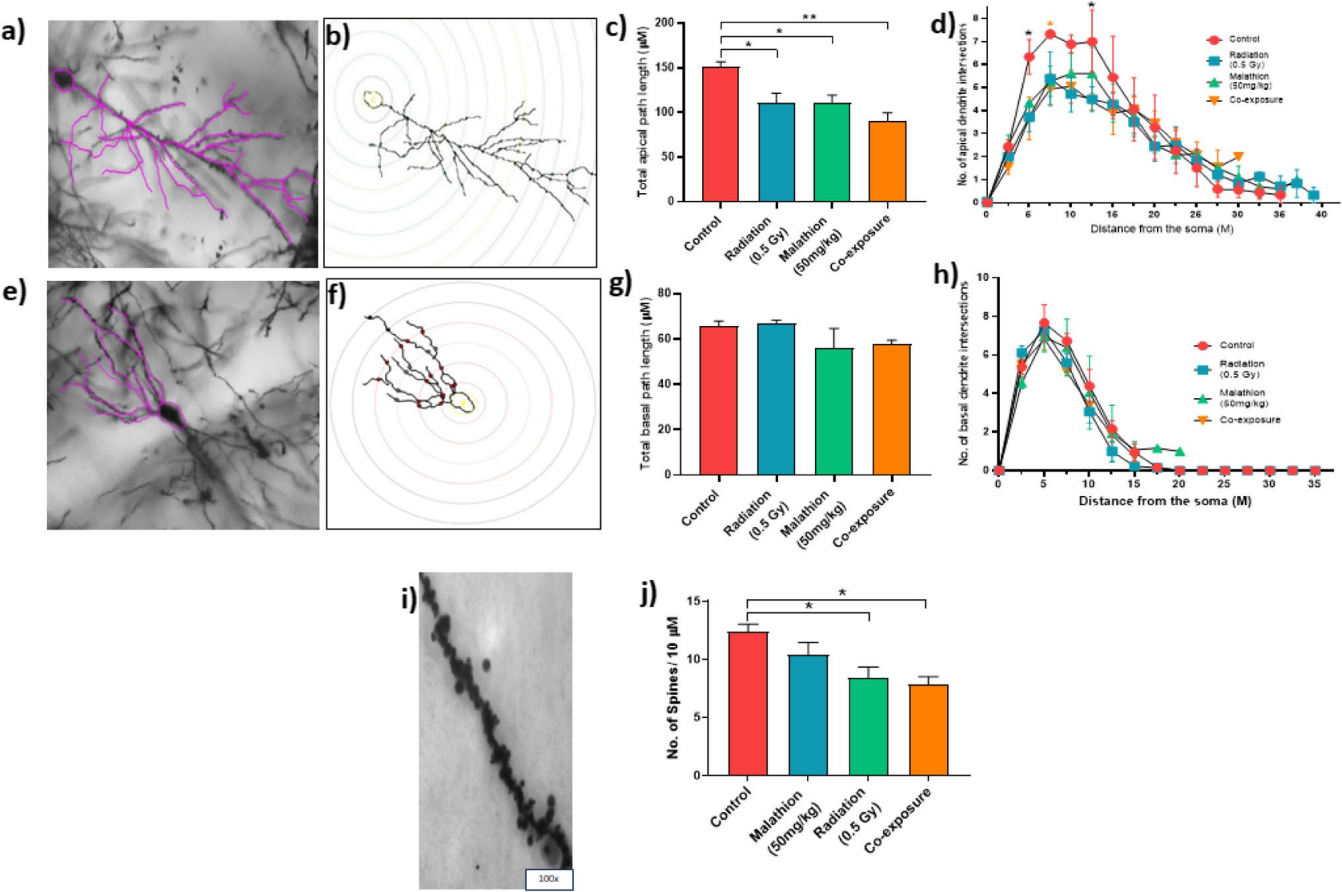
Effect of IR and malathion single and coexposure on neuronal morphology, dendritic arborization and spine density. (**a**) Representative image of traced apical dendrite of a pyramidal neuron (20 ×); (**b**) Representative image of an apical dendritic neuron after undergoing Sholl analysis; (**c**) Graph depicting total apical dendritic path length; (**d**) Graph depicting the number of intersection in the apical dendrites; (**e**) Representative image of traced basal dendrite of a pyramidal neuron (20 ×); (**f**) Representative image of a basal dendritic neuron after undergoing Sholl analysis; (**g**) Graph depicting total basal dendritic path length; (**h**) Graph depicting number of intersections in the basal dendrites; (**i**) Representative images of dendritic spines (100 ×); (**j**) Graph depicting the change in spine density between different groups (*n* = 3, *p* < *0.05, **0.01, ***0.001, data represented as ± SEM)

Neuronal tracing and Sholl analysis of apical (Fig. 2a and b) and basal dendrites (Fig. 2e and f) were carried out separately and depicted. Compared to the control, apical dendritic path length showed a decreasing trend in the IR (*p* < 0.05), malathion (*p* < 0.05), and coexposure (*p* < 0.01) groups (Fig. 2c). Furthermore, a decrease in the third intersection in the IR and malathion group, 4^th^ intersection in the coexposure group, and 6^th^ intersection in the IR and coexposure groups compared to the control was observed in the number of apical intersections (Fig. 2d) (*p* < 0.05). However, no changes were observed in the total basal dendritic path length and number of basal intersections (Fig. 2g and h). Furthermore, both the IR and coexposure groups showed a decrease in dendritic spine density (Fig. 2i and j) (*p* < 0.05). Therefore, the results indicate that IR and malathion coexposure further impede synaptic transmission and impaired plasticity as the highest decrease in the total apical path length and the highest number of dendritic spine loss was seen compared to a single exposure.

### Antioxidant capacity

**Fig. 3:**
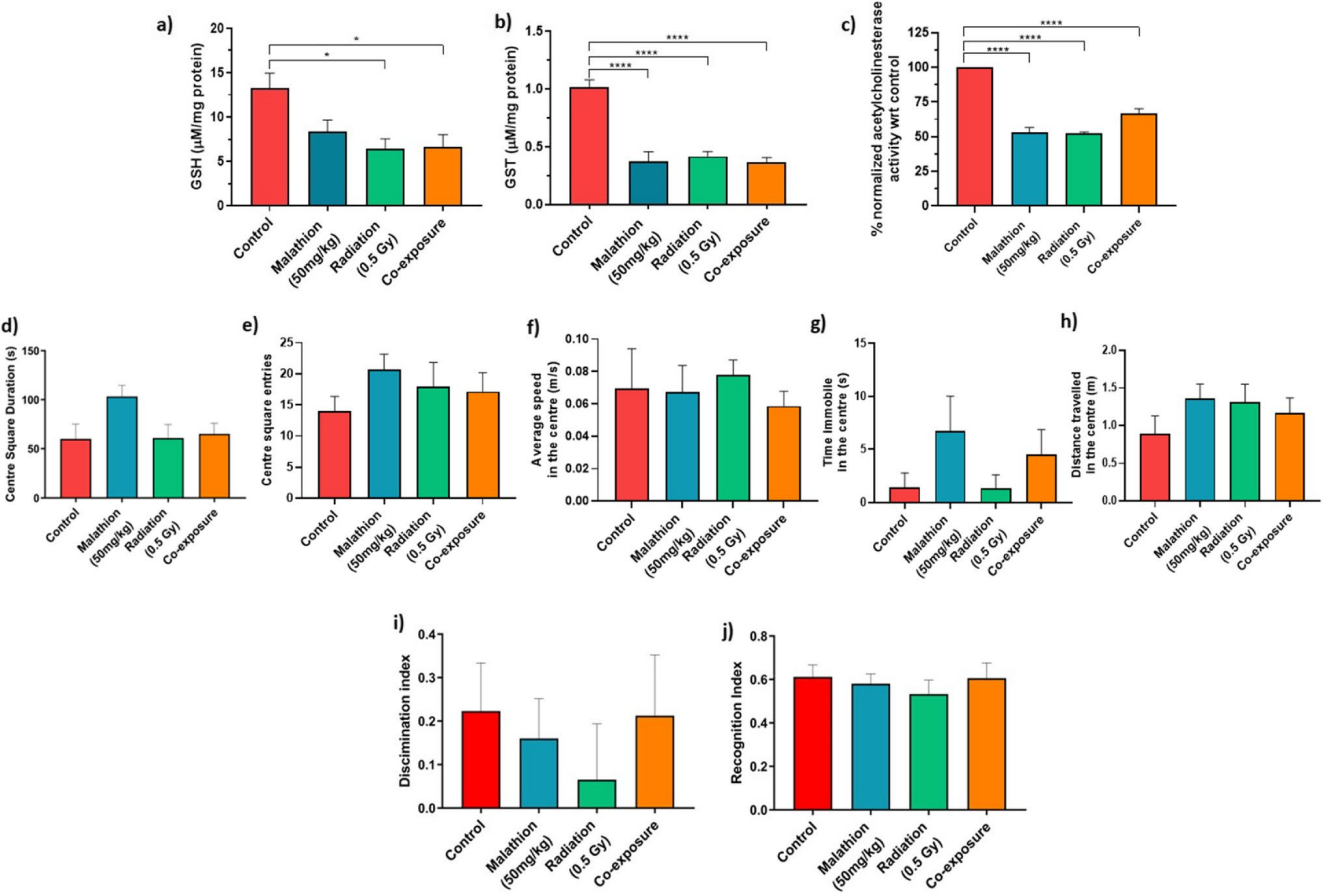
Effect of IR and malathion single and coexposure on enzymes and behaviour. Graphs depicting (**a**) GSH levels between groups; (**b**) GST levels between groups; (**c**) % normalized acetylcholinesterase enzyme inhibition activity between groups (*n* = 6, *p < 0.05, ****0.001, data represented as ± SEM). Graphs depicting (**d**) Centre square duration in the open field test; (**e**) Centre square entries in the open field test; (**f**) Average speed in the centre in the open field test; (**g**) Time immobile in the centre in the open field test; (**h**) Distance travelled in the centre in the open field test; (**i**) Determination index in novel object recognition; (**j**) Recognition index in novel object recognition (*n* = 9) (data are represented as mean ± SEM)

GSH levels were lower in the IR and coexposure groups than in the control group (*p* < 0.05) (Fig. 3a). Similarly, GST levels were also lowered in all three treatment groups with respect to the control (*p* < 0.001) (Fig. 3b). IR and coexposure showed comparable GSH levels, followed by malathion. On the other hand, malathion and coexposure showed comparable GST levels followed by IR. Therefore, IR and malathion interact to form a non-additive effect in antioxidant capacity.

### AChE inhibition

The average AChE activity of control samples was 1.039 ± 0.050 µmol/min/mg, malathion 0.549 ± 0.0497 µmol/min/mg, radiation 0.544 ± 0.040 µmol/min/mg and for coexposure was 0.694 ± 0.046 µmol/min/mg and are expressed as % normalized activity with respect to control**.** The levels of acetylcholinesterase were significantly reduced in all three treatment groups (*p* < 0.001) (Fig. 3c), indicating that IR and malathion treatment causes excitotoxicity through the buildup of acetylcholine. While the levels of individual exposures were comparable, coexposure did not display any significant changes compared to individual exposure.

### Behavioural changes

All three treatment groups, IR, malathion, and coexposure, did not show any significant changes in exploratory behaviour when scored for parameters like centre square entries, time immobile in the centre, average speed in the centre and distance travelled in the centre (Fig. 3d–h). No change was observed in the discrimination or recognition index upon introducing the novel object to the box in any treatment group (Fig. 3i and j). Therefore, single and combined malathion and IR exposure did not cause acute behavioural changes.

### Transcriptomic analysis

**Fig. 4:**
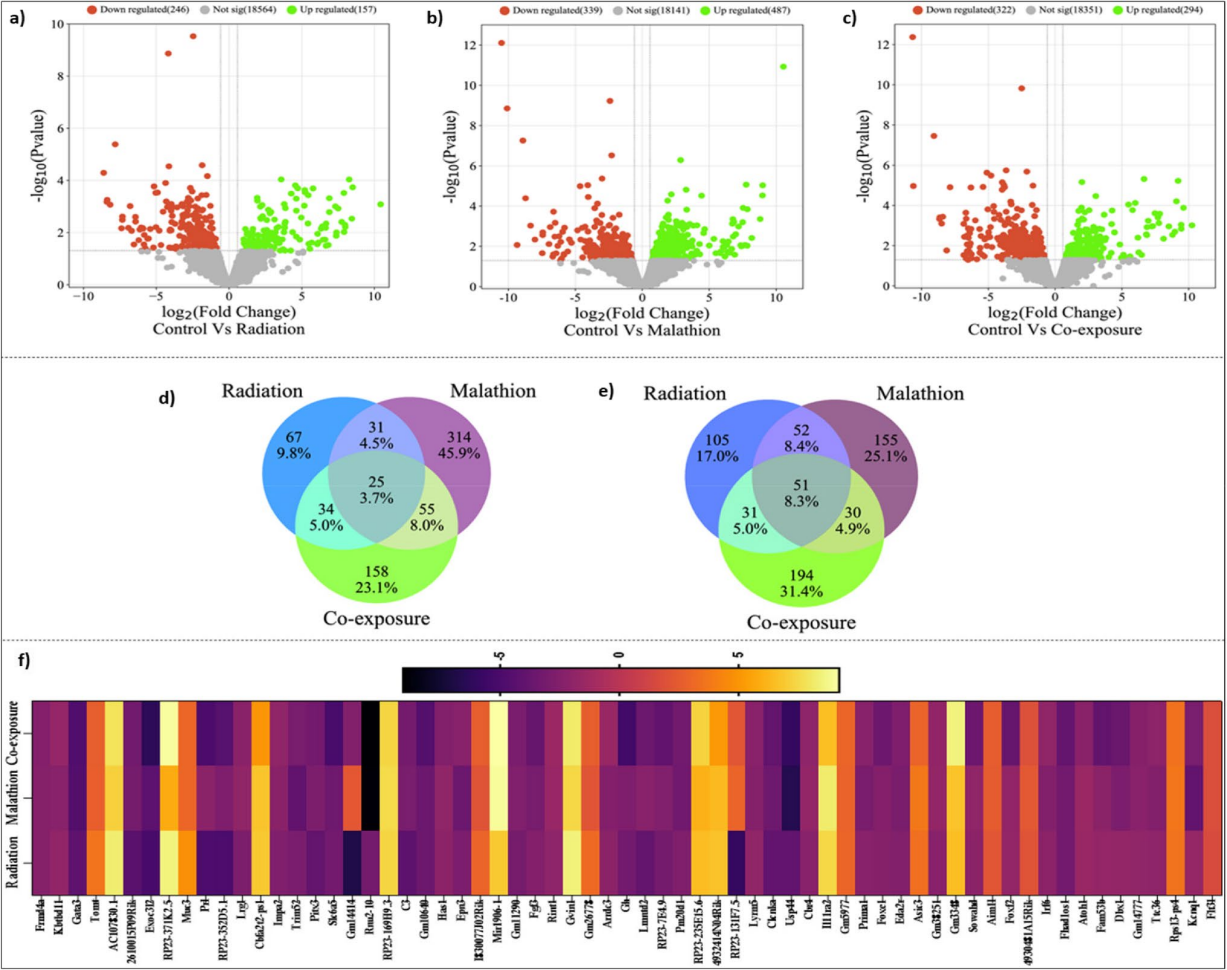
Differentially expressed genes post-exposure unique and common to IR, malathion and coexposure. Volcano plot showing the number of significant DEGs in the (**a**) IR (**b**) Malathion (**c**) Coexposure groups. Venn diagram displaying the number of DEGs in all three groups: (**d**) Upregulated, (**e**) Downregulated; (**f**) Heatmap depicting the number of commonly dysregulated genes in all three groups and their respective expression pattern expressed in terms of fold change value (*n* = 2, *p* < 0.05 values considered significant)

**Fig. 5:**
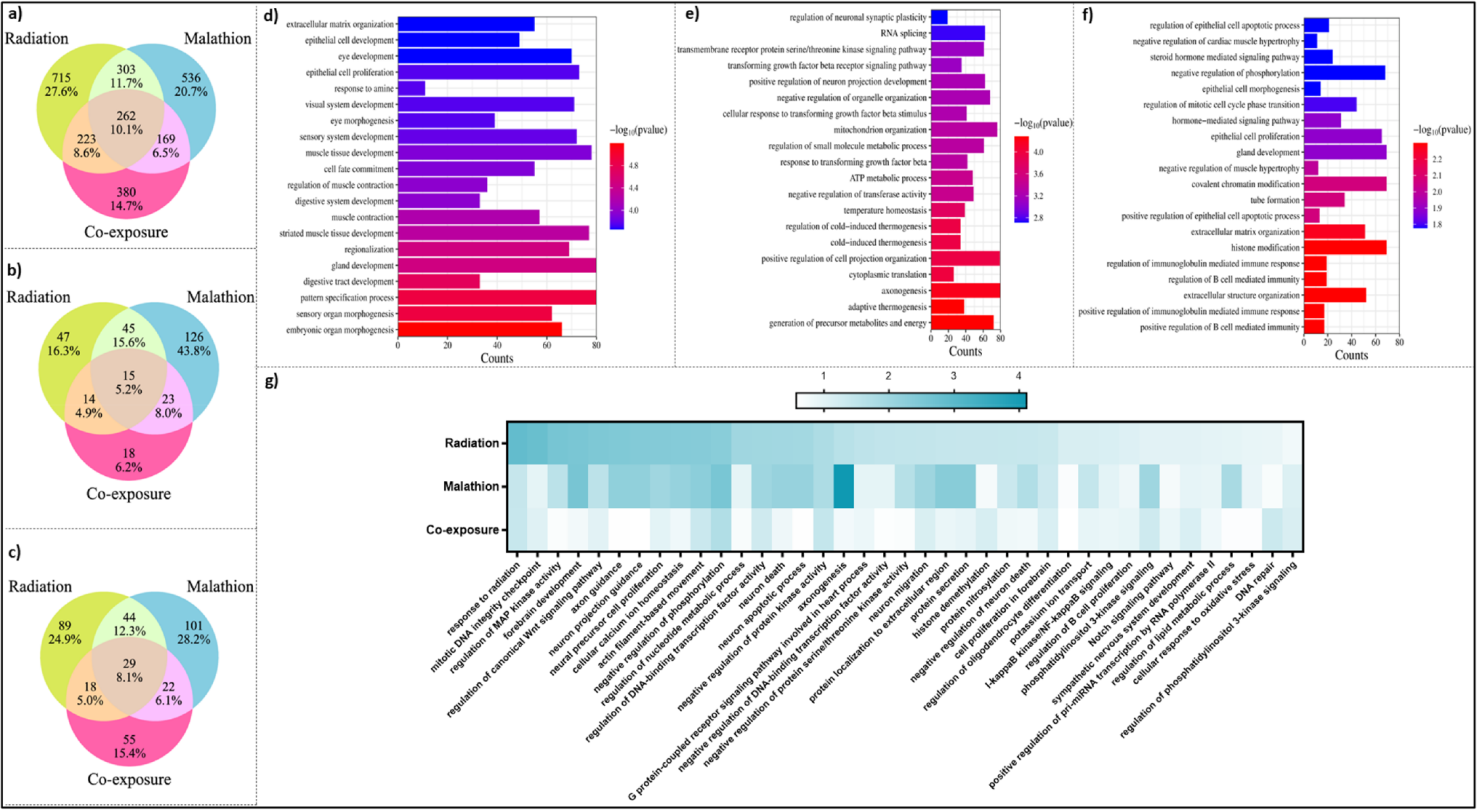
Biological processes related to neuronal survival and functioning altered post-IR, malathion and combined exposure. Venn diagram displaying the number of (**a**) Biological processes altered in all three groups, (**b**) Cellular components altered in all three groups (**c**) Molecular functions altered in all three groups; Top 20 enriched biological processes affected in (**d**) IR (**e**) Malathion (**f**) Coexposure (**g**) Selected common processes in all three groups and their enrichment value represented as a heatmap (*n* = 2, *p* < 0.05 values considered significant)

Differential expression analysis revealed 18,967 differentially expressed genes (DEGs) with respect to the control, out of which 246 genes were downregulated and 157 genes were upregulated in the IR (Fig. 4a). In the malathion treatment group, 339 genes were downregulated, and 487 genes were upregulated (Fig. 4b), while in the coexposure group, 322 genes were downregulated and 294 genes were upregulated (Fig. 4c) (supplementary Excel sheet 1). Twenty-five genes were commonly upregulated between all three treatment groups (Fig. 4d), and 51 genes were commonly downregulated between all three treatment groups (Fig. 4e). After filtering the genes according to the threshold, 65 genes in total were found to be common between the IR, malathion, and coexposure groups, and these DEGs and their respective fold change variation between the three different groups were represented in a heatmap (Fig. 4f). Gene ontology (GO enrichment) revealed that 262 common biological processes (BPs), 15 common processes (CCs), and 29 common molecular functions (MFs) were affected between the IR, malathion, and coexposure groups. 303 BPs, 45 CCs, and 44 MFs were common between the IR and malathion groups (Fig. 5a–c). The functionally enriched protein–protein interaction (PPI) network for the altered DEGs in different groups is depicted in Supplementary Figs. 1–3. A smaller number of enrichment values indicates the less random the occurrence of the genes within the network and the more significant their association.

#### IR single exposure

**Fig. 6:**
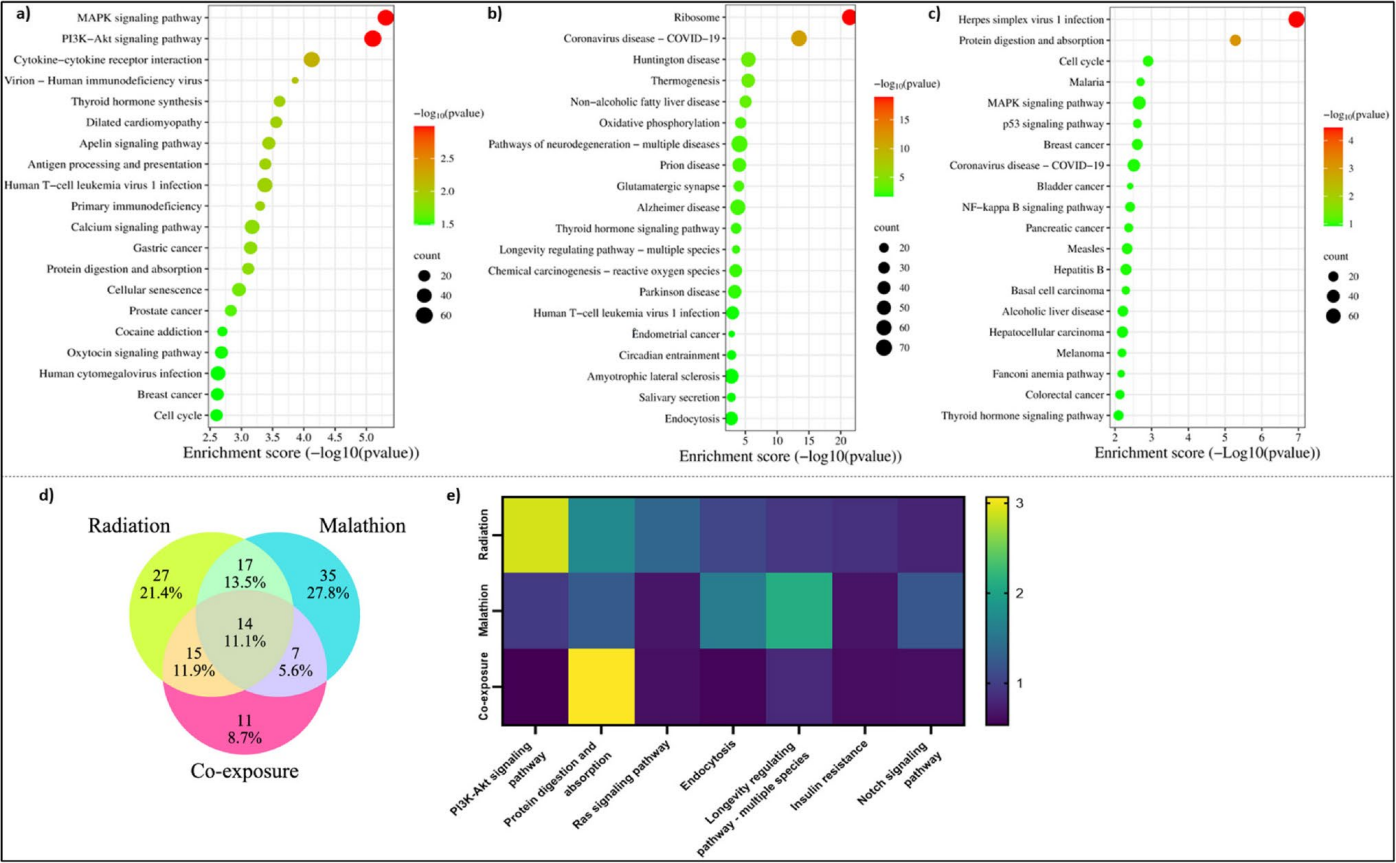
Pathways altered post single and combined exposure to IR and malathion reveal common pathways regulating neuronal differentiation, development and survival. Top 20 enriched pathways affected by (**a**) IR (**b**) Malathion (**c**) Coexposure; (**d**) Venn diagram displaying the number of common and unique pathways altered in all three groups (**g**) Selected common pathways altered in all three groups and their respective enrichment values represented as a heatmap (*n* = 2, *p* < 0.05 values considered significant)

GO enrichment showed that 1503 BPs were affected; among these, the top twenty altered biological processes included extracellular matrix organization, epithelial cell development, proliferation, response to amine, sensory system development, etc. (Fig. 5d). Seventy-three pathways were significantly affected in the IR group. Some of the major pathways, such as the MAPK, PI3K-Akt, apelin, NF-κB, TGF-β, cAMP and Notch signalling pathways, cholinergic synapse, serotonergic synapse, dopaminergic synapse and axon guidance, etc., which play an important role in regulating neuronal function and survival, were altered (Fig. 6a). The STRING PPI of the IR group had 225 nodes and 32 edges with a PPI enrichment value of 0.00332 (Supplementary Fig. 1). Genes encoding for inducing immune response and protein transcription formed a significant cluster, while the rest of the network was limited to two–three gene interactions.

#### Malathion single exposure

GO enrichment revealed that 1270 biological processes were affected, among which the top twenty biological processes were regulation of neuronal synaptic plasticity, RNA splicing, mitochondrion organization, axonogenesis, etc. (Fig. 5e). Seventy-three pathways were affected in the IR group. Some of the major pathways, such as the ribosome, Huntington’s disease, oxidative phosphorylation, glutamatergic synapse, AD, PD, ALS, Notch signalling pathway, and axon guidance, which play an important role in neurodegenerative pathogenesis, were altered (Fig. 6b). The STRING PPI network of malathion had 446 nodes and 676 edges with a PPI enrichment value of 1^–16^ (Supplementary Fig. 2). The major interacting network comprised genes encoding for ribosomal proteins, proteolytic degradation, and protein transcription.

#### Coexposure

GO enrichment revealed 1034 BPs that were affected, which included regulation of epithelial cell apoptotic process, regulation of immunoglobin-mediated immune response, histone modification, etc. (Fig. 5f). A total of 380 BPs were unique to the coexposure group, of which 223 BPs were common between the IR alone and coexposure groups, and 169 BPs were common between the malathion and coexposure groups (Fig. 5a). Fourteen pathways were common between all three groups. Some of the major signalling pathways, such as MAPK, p53, NF-κB, JAK-STAT, Notch, Rap1, Hippo, FoxO, and PI3K-Akt, which play an important role in the immune response, neuronal proliferation and survival, and synaptic plasticity, were altered (Fig. 6c). The STING PPI network of the coexposure group had 341 nodes and 97 edges with a PPI enrichment value of 0.000558 (Supplementary Fig. 3). Like malathion, a network cluster of genes encoding for protein transcription RNA binding was affected.

## Discussion

The role of IR and pesticides in neurotoxic symptoms is well known. However, limited information is available on the molecular mechanism behind the effect of low-dose IR or pesticides and how early exposure can contribute to neurotoxicity. The present study aimed to elucidate the mechanism involved in the manifestation of neurotoxic and subsequent neurodegenerative effects post coexposure to IR and malathion.

One of the earliest cellular responses to neurotoxic stimuli involves the initiation of neuroinflammation, typically through the activation of resident microglia and astrocytes, which constantly monitor the brain microenvironment for the presence of damaged or dead cells, contribute to synaptic plasticity and maintain homeostasis (Gelders et al. 2018). Studies have reported that IR induces a decline in hippocampal neurogenesis and neuroinflammation through increased pro-inflammatory markers such as Iba-1, Cd68, and Cd11c (single whole-body exposure of 0.1 and 2 Gy) (Acharya et al. 2015). Furthermore, levels of pro-inflammatory markers like IL-6 and TNF-α have been shown to rise post-malathion treatment (100 mg/kg bw) (Mohammadzadeh et al. 2018). Similarly, in the present study, reactive astrogliosis was increased in IR and coexposure, indicating immune response.

In the present study, inhibition of acetylcholinesterase enzyme activity was observed in the IR and malathion treatment groups. Previously, only at a higher dose (100 mg/kg bw), malathion has been linked to AChE inhibition (Trevisan et al. 2008); however, studies looking at the effect of IR on the same are sparse. X-ray exposure generates ROS, which can damage the structure and function of proteins and induce oxidative stress. Oxidative stress is linked to the inhibition of AChE activity (Vecchio et al. 2021). Further, DNA damage caused by X-rays can activate signalling pathways that inhibit AChE activity (Thangaraj et al. 2016). In addition, radiation can disrupt normal signalling pathways, disrupting the synthesis, transport, or stability of proteins like AChE. Our study findings have also demonstrated that radiation causes oxidative stress that could lead to acetylcholinesterase inhibition. Further, as expected, we noticed AChE inhibition in malathion groups, as it is one of the known mechanisms of action. However, we saw slightly reduced AChE inhibition levels in coexposure compared to individual exposure, possibly due to unknown antagonistic interaction. We further noted a decrease in GSH in the IR and coexposure groups and reduced levels of GST in all three treatment groups. Similar results were seen with malathion administration in rats (Trevisan et al. 2008). The involvement of redox machinery and the detoxification of free radicals by antioxidant enzymes have been previously linked to neurodegeneration (Nakamura et al. 2012), indicating that single or combined exposure to malathion and IR can induce similar effects.

In the present study, the pyknotic cells were higher in both the malathion and IR singular and coexposure groups. Neuronal viability in the brain is closely associated with conditions involving tissue injury or neurodegeneration (Morrison et al. 2002) and is also one of the key hallmarks of neurodegenerative diseases (Hirsch 1999). Previously, IR has been shown to induce cell death in human neuronal progenitor cells (Katsura et al. 2016,) and malathion has also been associated with reduced neuronal survival (Abdel-Rahman et al. 2010). Furthermore, percentage NeuN reactive area indicating mature neurons reduced in the malathion, IR, and coexposure groups in the DG region. Interestingly, out of all three treatment groups, the coexposure group showed the highest number of pyknotic cells and the lowest number of viable mature neurons, which indicates that the combined treatment of malathion and IR produced an overall synergistic effect on the neurons. Similar phenomena with both synergistic and antagonistic effects have been shown to occur whenever environmental chemicals and other stressors have been known to combine (Holmstrup et al. 2010). Varied interactions may be seen when individual chemicals with similar or dissimilar modes of action may affect the toxicity of each other either through synergism or addition or antagonism depending on the dose and time window of the interaction (Silins and Högberg 2011) or show completely different effects than observed singularly. In our study, although astroglial activation in coexposure was not the highest compared to IR, oxidative damage and cell death showed synergistic effects, while the percentage of acetylcholinesterase enzyme inhibition activity with respect to the control was at closely comparable levels in the malathion and IR groups, showing a slight antagonistic effect. This finding affirms that the effect of the interplay of various neurotoxicants is contingent upon the dose, duration, and physiological variations within the exposed individual, making it difficult to predict the response. Therefore, increased oxidative stress, increased neuroinflammation, decreased viability, and increased pyknotic cells may devastate neuronal homeostasis.

Dendrites and dendritic spines are crucial in synaptic transmission, controlling behaviour and memory, and helping in structural remodelling during synaptic plasticity (Calverley and Jones 1990). They are vital in processing large synaptic inputs, and their reduction may impair neuronal transmission and lead to their eventual death (Segal 2010). Singular and combined exposure to malathion and IR contributes to neuronal dysfunction by inducing changes in dendritic morphology, arborization, and dendritic spine density. A search of studies on dendritic morphology analysis post malathion exposure yielded only one other study where malathion (40 mg/kg, intraperitoneal) showed altered dendritic arborization and spine density (Wang et al. 2020), while IR also yielded similar results at 0.1 and 1 Gy (Parihar et al. 2015). It has been speculated that dendritic spine changes are markers of early neurodegeneration (Lin et al. 1997).

### Malathion exposure activates distinct neuronal processes and neurodegenerative pathways

Transcriptomic studies can shed light upon crucial changes on a genome-wide level and provide valuable insights into the modulation of various cellular processes and altered signalling pathways. To date, there are no reports on the transcriptional regulation of malathion-induced neurotoxicity. Malathion exposure altered several biological processes, such as axonogenesis, axon guidance, neuron differentiation, neuron death, regulation of neuronal synaptic plasticity, regulation of nervous system processes, synapse organization, calcium ion homeostasis, regulation of neurotransmitter levels, neural precursor cell proliferation, regulation of postsynaptic membrane potential, dendrite and dendritic spine development, negative regulation of neurogenesis, gliogenesis, and neuron cellular homeostasis. These changes directly correlated with the histological changes observed in neuronal morphology, neuronal plasticity, neuroinflammatory response, and neuronal cell death. Malathion treatment in rodents has previously been associated with altered dendritic morphology (Campaña et al. 2008), neuroinflammation, apoptotic cell death, and cognitive changes (dos Santos et al. 2016). Several crucial processes affecting mitochondrial functioning were also altered, such as the ATP metabolic process, mitochondrion organization, mitochondrial respiratory chain complex assembly, and oxidative phosphorylation. Oxidative phosphorylation is a key pathway involved in neurodegeneration (Chan 2020), and malathion has previously been shown to induce mitochondrial dysfunction (dos Santos et al. 2016; Venkatesan et al. 2017). Further, mitochondria are implicated in causing neurodegeneration (Subramaniam and Chesselet 2013).

We observed alterations in several neurodegenerative pathways related to AD, PD, Huntington’s disease, and ALS. Previous studies have shown that malathion induced several changes in the brain comparable to AD phenotype and was a good model for studying AD (Venkatesan et al. 2017). Furthermore, pathways affecting related neuronal processes, such as glutamatergic synapses, axon guidance, spinocerebellar ataxia, and GABAergic synapses, were also altered. Among the key signalling pathways affected, the Notch, AMPK, PI3K-Akt, apelin, Wnt, calcium, and cAMP signalling pathways were a few altered pathways. These pathways have been shown to have a significant influence on the pathogenesis of neurodegenerative diseases (Domise and Vingtdeux 2016; Saito et al. 2019; Ureshino et al. 2019; Di Benedetto et al. 2021; Razani et al. 2021); however, they have not explicitly been studied for their involvement in malathion-induced neurotoxicity. Interestingly, some of these pathways, such as the Notch, Wnt, and apelin signalling pathways, have been reported to be neuroprotective by modulating neurogenesis and synaptic plasticity (Arrázola et al. 2015; Zhu et al. 2019; Ho et al. 2020). Remarkably, the most abundantly altered pathway in our study involved the ribosome and several ribosomal protein-coding genes whose role has not been studied concerning malathion exposure and is less explored in neurodegeneration studies. Several ribosomal, protein transcription and translation processes, processes related to DNA damage and repair, cell signal activation and transport, which included genes such as Rpl41, Rpl14, RP19, Rp19-ps6, Rps13, Rps10-ps1, and Rpl17, which encode ribosomal proteins involved in large and small ribosomal subunit biogenesis, translation and RNA binding, were affected. Studies have shown that proper ribosomal synthesis, continued mRNA translation, and protein production are critical for maintaining the dendritic tree, dynamic proteome remodelling, synapse formation, axon growth, and synaptic plasticity, and impairment can cause disconnection of neuronal circuitries (Slomnicki et al. 2016; Dastidar and Nair 2022). Furthermore, ribosomal translational dysregulation is among the important events leading to the pathogenesis of disorders such as AD and PD (Ding et al. 2005). Therefore, targeting the ribosomal pathway could be a novel approach to mitigate organophosphate toxicity and hence needs further research. Most of these findings have not been previously reported to be associated with malathion exposure and thus aid in further understanding malathion’s noncholinergic mechanism of action.

### IR exposure results in altered neurotransmitter processes and modulation of neuronal growth, survival, and death

Singular exposure to IR altered major biological processes controlling neuronal functioning, such as axon guidance, regulation of neural precursor cell proliferation, positive regulation of long-term synaptic potentiation, calcium ion homeostasis, neuron apoptotic process, regulation of neuron death, dopamine metabolic process, axonogenesis, synaptic transmission, nerve growth factor signalling pathway, gliogenesis, neurotransmitter loading into a synaptic vesicle, dopamine receptor signalling pathway, astrocyte differentiation, neurotransmitter uptake, dopaminergic neuron differentiation, and many more related processes. Interestingly, many dopaminergic processes are altered, which plays a crucial role in disorders such as PD (Michel et al. 2016). Many cell signalling processes, such as regulation of MAP kinase activity, cAMP-mediated signalling, regulation of canonical Wnt signalling, ER-nucleus signalling, nerve growth factor signalling, positive regulation of phosphatidylinositol 3-kinase signalling, intrinsic apoptotic signalling, I-kappaB kinase/NF-κB signalling, Notch signalling, Hippo signalling, and Toll-like receptor signalling, etc., were altered. The affected processes are involved in the regulation of the cellular response to damage through the induction of inflammatory pathways, production of neurotrophins to promote neurogenesis and neuronal survival, induction of the cellular stress response, and repair initiation and neuroprotection, which correlate with our earlier histological observations. Previously, Kempf et al. (2016) noted changes in synaptic plasticity, neuronal degeneration, Wnt/β-catenin, and glutamate receptor signalling at 0.3 Gy. Some of the prominent pathways that were enriched following IR exposure included MAPK, PI3K-Akt, apelin, cCalcium, NF-κB, TGF-β, Rap1, chemokine, cAMP, p53, and Notch signalling pathways, serotonergic synapse, axon guidance, cholinergic synapse, tyrosine metabolism, and dopaminergic synapse, etc. Most of the pathways affected were similar to malathion exposure; however, while glutamatergic and GABAergic synapses were affected in malathion, in IR exposure, serotonergic and dopaminergic synapses, which are well-known major targets of neurodegeneration (Lowe et al. 2016), were altered. Studies have previously reported similar changes in processes related to neuronal synaptic plasticity, axonal guidance, and altered signalling pathways such as cAMP and ERK/MAPK but also noted glutamate receptor signalling changes similar to the pathogenesis of Alzheimer’s in patients. Some of these processes have been previously reported to be altered in response to IR (Veeraraghavan et al. 2011; Kempf et al. 2015). However, many of the signalling processes that have been uniquely altered in our present study have thus far been reported to be altered only in high-dose IR scenarios, implying that there is a dearth of information yet to be unearthed in this aspect.

### Coexposure activates pathways regulating neuronal cell fate, differentiation, development, and survival

An analysis of the commonly affected biological processes altered between single and combined exposure revealed several processes that regulate neuronal functioning, such as axon guidance, neuron projection guidance, neural precursor cell proliferation, neuron apoptotic processes, axonogenesis, several protein kinase-mediated signalling processes, and the DNA damage and repair response. Fold enrichment showed downregulation of these processes in coexposure compared to singular exposure to IR and malathion, indicating that malathion and IR interact and can inhibit gene expression. Similarly, some of the pathways, such as the PI3K-Akt, Ras, and Notch signalling pathways, were notable common alterations wherein, compared to individual exposure, were downregulated in coexposure, again implying that coexposure can have different detrimental effects compared to the individual exposure. Some of the signalling pathways, such as the Hippo, FoxO, and JAK-STAT signalling pathways, were found to be uniquely altered only in the coexposure groups. While FoxO, belonging to a family of transcription factors, plays a role in the stress response, neuronal signalling, and survival (Santo and Paik 2018), the JAK-STAT pathway and Hippo signalling have been shown to activate microglia-induced neuroinflammatory response and promote apoptosis (Qin et al. 2016; Sahu and Mondal 2020). Other notable pathways like cytokine‒cytokine receptor interaction, PI3K-Akt signalling pathway and neuroactive ligand-receptor interaction pathway were altered. As mentioned previously, dysregulation of the PI3K-Akt pathway can hinder normal neuronal functioning, while cytokine activation and release are commonly associated with neurodegenerative disorders (Wang et al. 2015). When the unique processes altered in different groups were analysed, the IR group showed many DNA repair and catecholamine processes to be majorly altered. In contrast, the malathion group showed changes majorly related to genes involved in ribosomal protein functions, protein transcription,translation and mitochondrial dysfunction. Coexposure altered both genes of DNA replication, repair, protein processing, and several genes affecting developmental processes. Rnu2-10 (U2 small nuclear RNA 10), which can impair motor function in animals, Exoc3l2 (exocyst complex component 3-like 2) important for vasculature, Gh (growth hormone) involved in neurogenesis and synaptogenesis, Slc6a5 (solute carrier family 6 (neurotransmitter transporter, glycine), member 5) which is a glycine transporter were among the most downregulated in coexposure compared to individual exposure. At the same time, the expression of upregulated genes was almost comparable between the three groups.

Therefore, therapeutic interventions involving the inhibition of these pathways could aid in mitigating neurotoxicity during exposure scenarios. The PPI network in the coexposure group revealed changes similar to malathion, where the cluster with highly enriched genes included genes such as Rpl28, Gm9493, Rps13, Rps27rt, etc., involved in functions such as RNA translation and export and coding for ribonuclear proteins. Like IR, the rest of the clusters contained approximately 3–4 genes controlling various processes. Therefore, similar to malathion, the ribosome was the major cellular component affected by coexposure, indicating that the influence of malathion was greater than that of IR.

Previously, IR has been known to induce impairment in spontaneous behaviour, spatial memory, and logical reasoning and cause learning deficits (single dose of 0.5 Gy of ^1^H and 0.1 Gy of ^1^6O) (Howe et al. 2019). In the present study, although several pathways related to neurons were altered, neither the open field test nor novel object recognition for the assessment of exploratory behaviour, anxiety, memory, and recognition ability revealed any significant changes, suggesting that these molecular alterations may not have manifested in a functional capacity. It is also possible that any associated functional changes would have occurred after a latency period, but since our study focused at the early brain response to malathion or IR exposure, significant differences would not have been observed. Furthermore, it is possible that other behavioural changes, such as social behaviour, fear-induced memory or spatial memory, etc., might have been affected, which was not investigated in the present study. Hence, looking at lower doses of long-term chronic effects of both could aid us in understanding if the damage persists in repeated or single-exposure settings.

## Conclusion

To conclude, single or combined exposure to malathion and IR involves majorly overlapping biological processes and pathways affecting neuronal functions, which could be why they are both implicated in the etiology of neurodegenerative disorders. Our study provides new and detailed insights into the molecular modulation of neurotoxicity associated with malathion and IR exposure. It stresses the importance of minimizing exposure to organophosphates such as malathion in domestic settings or undergoing routine procedures involving unnecessary irradiation. Further, we have shown that exposure to neurotoxicants at a younger age can trigger the activation of neurodegenerative-like symptoms, which could cause detrimental effects to an individual later in life. Overall, our study suggests that exposure to neurotoxic agents such as malathion or radiation at a young age, when there are a greater number of developing cells sensitive to xenobiotic damage, could drive neuronal injury and cell death, thus propelling the occurrence of neurodegenerative complications in the exposed individual at an earlier pace than it usually would occur.

### Supplementary Information

Below is the link to the electronic supplementary material.

Supplementary file1 (XLSX 175 KB)

Supplementary file2 (XLSX 620 KB)

Supplementary file3 (XLSX 48 KB)

Supplementary file4 (DOCX 892 KB)

## Data Availability

The datasets generated during and/or analysed during the current study are available from the corresponding author on reasonable request.
