# Supplementary material for: Low-dose exposure to malathion and radiation results in the dysregulation of multiple neuronal processes, inducing neurotoxicity and neurodegeneration in mouse
Source: Environ Sci Pollut Res Int. 2023 Dec 1;31(1):1403–18. doi: 10.1007/s11356-023-31085-4 (PMC10789675; doi:10.1007/s11356-023-31085-4)
Supplement: Supplementary file 4 — Supplementary file4 (DOCX 892 KB) [file 11356_2023_31085_MOESM4_ESM.docx]

**Supplementary Figures:**


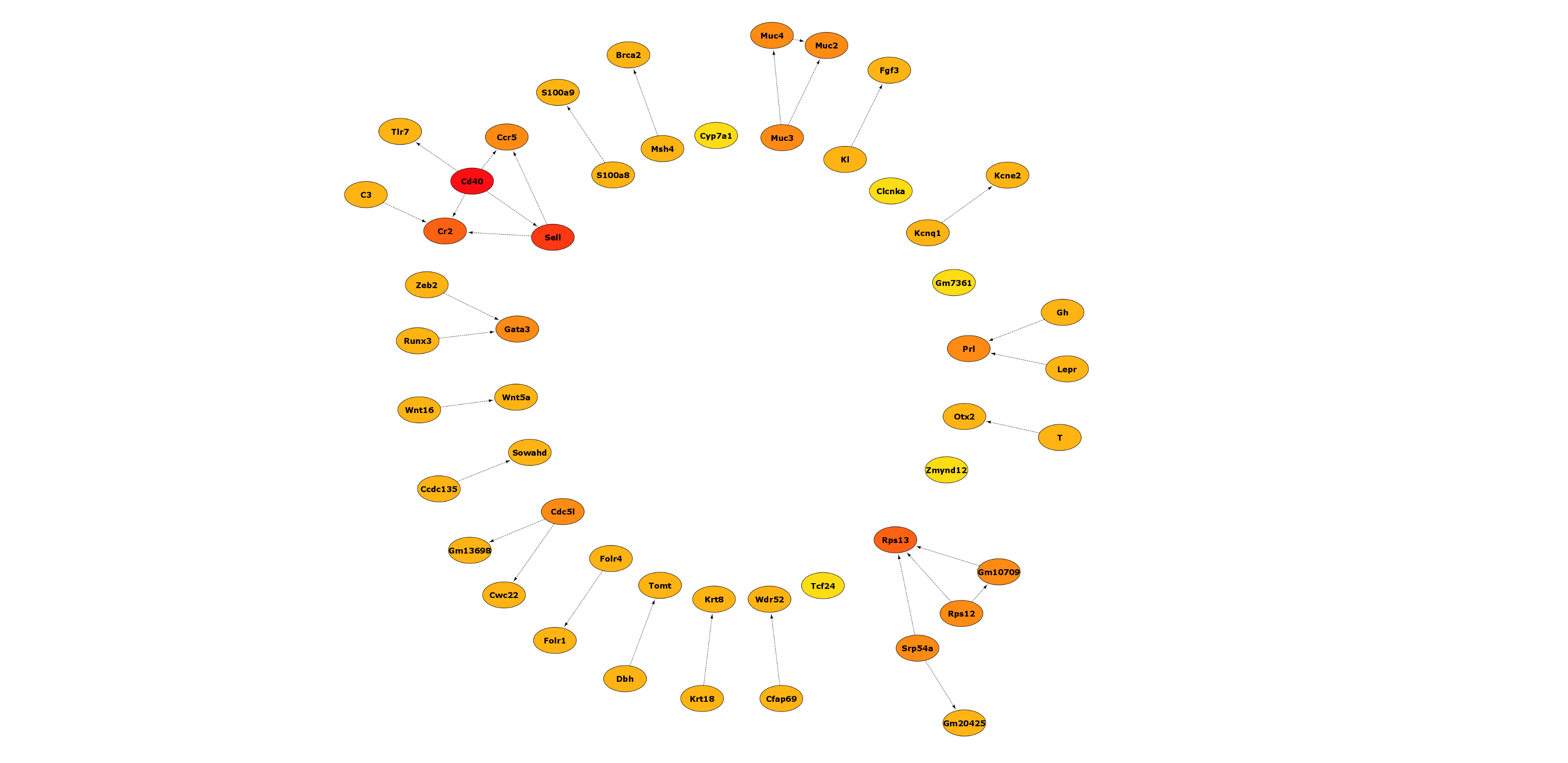


***Fig 1: STRING network depicting PPI interactions between genes post exposure to IR.*** *Functional protein‒protein interaction network of the DEGs of the IR group; a high confidence score of 0.7 was considered, the color intensity of the nodes represents the confidence of the interaction, and the arrows connecting the nodes detect the targets. Closely associated and interacting nodes are connected by lines (n=2, p<0.05 values considered significant).*


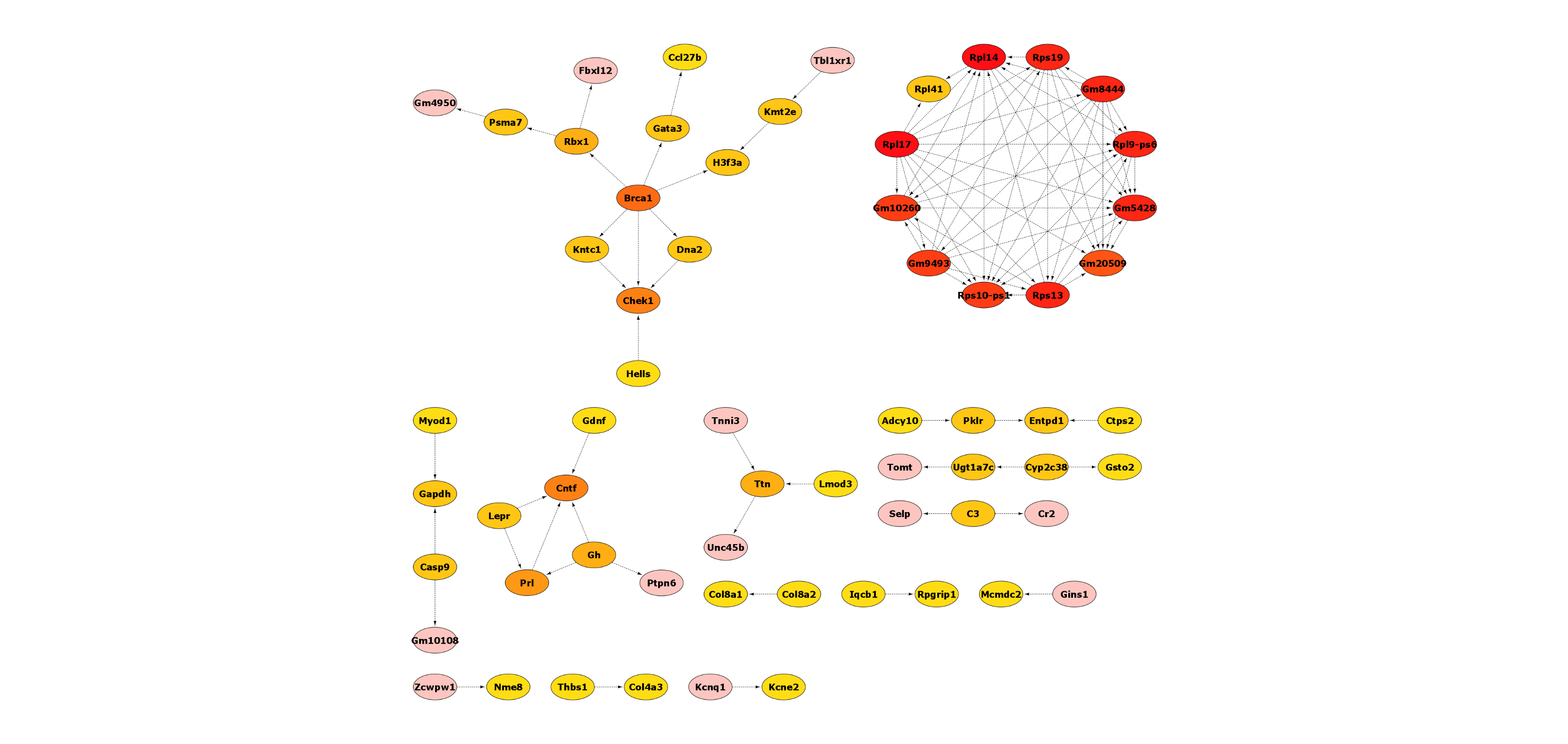


***Fig 2: STRING network depicting PPI interactions between genes post exposure to malathion.*** *Functional protein‒protein interaction network of the DEGs of the malathion group; a high confidence score of 0.7 was considered, the color intensity of the nodes represents the confidence of the interaction, and the arrows connecting the nodes detect the targets. Closely associated and interacting nodes are connected by lines (n=2, p<0.05 values considered significant).*


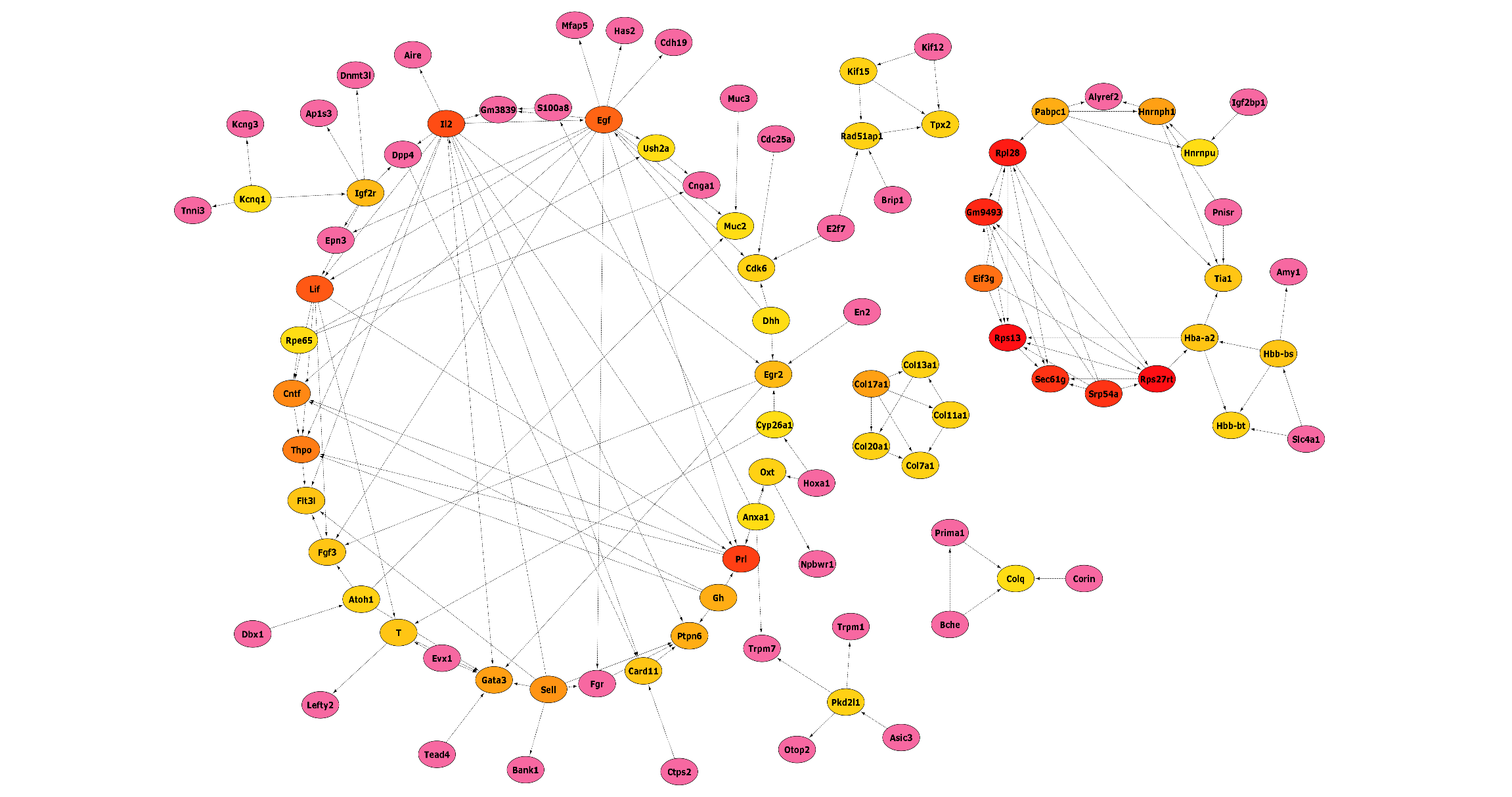


***Fig 3: STRING network depicting PPI interactions between genes post co-exposure.*** *Functional protein‒protein interaction network of the DEGs of the coexposure group; a high confidence score of 0.7 was considered, the color intensity of the nodes represents the confidence of the interaction, and the arrows connecting the nodes detect the targets. Closely associated and interacting nodes are connected by lines (n=2, p<0.05 values considered significant).*
